# Rare and Frequent Promoter Methylation, Respectively, of TSHZ2 and 3 Genes That Are Both Downregulated in Expression in Breast and Prostate Cancers

**DOI:** 10.1371/journal.pone.0017149

**Published:** 2011-03-14

**Authors:** Miyako Yamamoto, Emili Cid, Samuel Bru, Fumiichiro Yamamoto

**Affiliations:** 1 Burnham Institute for Medical Research (BIMR), La Jolla, California, United States of America; 2 Institute of Predictive and Personalized Medicine of Cancer (IMPPC), Badalona, Barcelona, Spain; SanfordBurnham Medical Research Institute, United States of America

## Abstract

**Background:**

Neoplastic cells harbor both hypomethylated and hypermethylated regions of DNA. Whereas hypomethylation is found mainly in repeat sequences, regional hypermethylation has been linked to the transcriptional silencing of certain tumor suppressor genes. We attempted to search for candidate genes involved in breast/prostate carcinogenesis, using the criteria that they should be expressed in primary cultures of normal breast/prostate epithelial cells but are frequently downregulated in breast/prostate cancer cell lines and that their promoters are hypermethylated.

**Methodology/Principal Findings:**

We identified several dozens of candidates among 194 homeobox and related genes using Systematic Multiplex RT-PCR and among 23,000 known genes and 23,000 other expressed sequences in the human genome by DNA microarray hybridization. An additional examination, by *real-time* qRT-PCR of clinical specimens of breast cancer, further narrowed the list of the candidates. Among them, the most frequently downregulated genes in tumors were NP_775756 and ZNF537, from the homeobox gene search and the genome-wide search, respectively. To our surprise, we later discovered that these genes belong to the same gene family, the 3-member Teashirt family, bearing the new names of TSHZ2 and TSHZ3. We subsequently determined the methylation status of their gene promoters. The TSHZ3 gene promoter was found to be methylated in all the breast/prostate cancer cell lines and some of the breast cancer clinical specimens analyzed. The TSHZ2 gene promoter, on the other hand, was unmethylated except for the MDA-MB-231 breast cancer cell line. The TSHZ1 gene was always expressed, and its promoter was unmethylated in all cases.

**Conclusions/Significance:**

TSHZ2 and TSHZ3 genes turned out to be the most interesting candidates for novel tumor suppressor genes. Expression of both genes is downregulated. However, differential promoter methylation suggests the existence of distinctive mechanisms of transcriptional inactivation for these genes.

## Introduction

Homeobox genes have a key role in the specification and patterning of body parts during development [1], [2], [3], [4], [5], [6], [7]. These genes contain a highly conserved 183-bp sequence (homeobox) and encode proteins that specifically bind to DNA acting as transcriptional modulators [8], [9]. There are at least 178 homeobox sequences in the human genome, 160 of which may be translated into homeodomains within functional proteins [10]. Whereas regulated cell growth and differentiation are the basis for development, cancer results from uncontrolled growth of undifferentiated cells. Accordingly, cancer may be regarded as a dynamic developmental disorder [11]. It is, therefore, not unreasonable to speculate that the activation/inactivation of certain homeobox genes may contribute to carcinogenesis. As a matter of fact, such examples have been reported. Activation of homeobox genes was described in hematopoietic cell lines either by incorporation of a viral regulatory element in the vicinity of the homeobox gene or by chromosomal rearrangement [12], [13], [14]. The oncogenic potential of certain deregulated homeobox genes was also demonstrated by using *in vitro* and *in vivo* transformation assays [15]. In contrast, other homeoproteins with tumor suppressor activity have also been reported [16], [17], [18], [19], [20], [21], [22].

We became interested in homeobox genes and its relationship with cancer after analyzing the result of a previous work using Methylation Sensitive-Amplified Fragment Length Polymorphism (MS-AFLP) fingerprinting [23]. MS-AFLP is an efficient and sensitive method that provides a rapid evaluation of DNA methylation alterations at *NotI* landmarks. Using this method we found that multiple homeobox and related genes exhibited alterations in band intensity in cancer. The first hypermethylated DNA fragment identified and characterized in most prostate and some breast, but not in colon, cancers contained a sequence from the HOX D Gene Complex, which is responsible for the morphogenesis of the genitoexcretory apparatus [24], [25], [26]. Additional fingerprints yielded other altered bands belonging to the same homeobox gene family. Although the MS-AFLP method provided an important clue to initiate the research on homeobox genes in cancer based on the alterations in DNA methylation, not all homeobox genes contain *NotI* sites.

Moreover, the relationship of DNA methylation status and cancer has several major concerns that must be clarified. An issue requiring resolution is if DNA methylation plays an active role in carcinogenesis, rather than a passive role. On one hand, DNA methylation alterations may occur progressively in cancer in a directional manner by activating those genes with oncogenic activity and/or inactivating those genes with tumor suppressor activity and maintaining actively those changes in cancer cells. On the other hand, DNA methylation changes may occur randomly, even under normal conditions, and the cells that happen to possess alterations which favor cell growth, may be selected during the course of carcinogenesis. There is also the possibility that DNA methylation is merely the result of carcinogenesis caused by an altered expression of DNA-methyltransferase(s), without any causal association with carcinogenesis. Other issues include aging-dependent DNA methylation alterations and the uncoupling of gene expression with the methylation state [27], [28]. DNA methylation patterns may change during aging, and this can happen to cancer and normal cells complicating the comparison among specimens belonging to different age groups. There are also changes in DNA methylation that do not result in alterations in gene expression, and therefore, their effects in carcinogenesis are negligible. Because gene expression is functionally more important than DNA methylation, we employed a more rigorous and logical approach to identify homeobox genes involved in carcinogenesis, by directly determining their expression.

## Results

**Figure 1:**
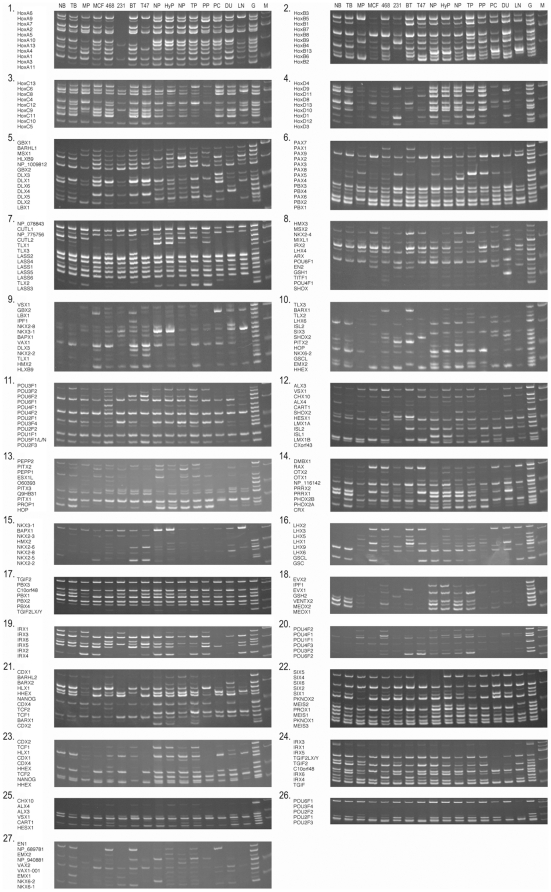
SM RT-PCR results of breast and prostate cells and tissues. The results of 27 sets of experiments are shown. cDNA sources are abbreviated as follows: normal sample (NB) and primary tumor (TB) of breast tissue from an individual; normal sample (NP), and primary tumor tissues (TP) of prostate from an individual; normal prostate tissue (NP) from a third individual; a hyperplastic prostate tissue (HyP) from a fourth individual; primary cultures of normal mammary (MP) and prostate (PP) epithelial cells; and MCF7 (MCF), MDA-MB-468 (468), MDA-MB-231 (231), BT-20 (BT), T-47D (T47), PC3 (PC), DU145 (DU), and LNCaP (LN) cancer cell lines. The genomic locations of the DNA fragments amplified from each individual gene are also shown on the left side of gel pictures. The symbol M denotes DNA fragment size markers, and G heads the results of control genomic DNA. Differential expression was observed with some homeobox genes.

We constructed a SM RT-PCR system to analyze the expression of homeobox genes. We designed two primers per gene to amplify DNA fragments of different sizes in single exons for the same gene group. For the 39 HOX genes in the HOX Gene Complexes, which exhibit significantly high sequence homology, primers were chosen for one conserved region sequence and another unique region sequence in order to prevent artificial recombinations among different gene transcripts [29]. For the other homeobox genes with lower sequence homology, we used two unique primers. The list of the genes is shown in Supplementary Table S1, together with the nucleotide sequences of primers, their concentrations used, and the sizes of the amplified DNA fragments. We were unable to design unique primer pairs to discriminate the X-linked and Y-linked TGIF2L genes, and therefore, both genes were amplified together. Additionally, PAX1, PAX2, PAX5, PAX8, and LASS1 genes were incorporated into the system because other members of their families possess a homeodomain although they did not present it themselves. Together with those 5 non-homeobox genes, the total SM RT-PCR system covered 194 (195 if TGIF2LX and TGIF2LY were counted separately) genes with some overlaps in 27 sets of multiplex reactions. Using the homeobox SM RT-PCR system, we examined the gene expression in normal and cancer cells of breast and prostate. We placed an emphasis on the comparison between primary culture of normal epithelial cells and established cancer cell line cells, circumventing the heterogeneity and contamination problems of tissues by the use of more homogeneous cells. Results are shown in Figure 1. We identified 3-dozen homeobox genes whose expression was altered in cancer cell line cells. They are listed in Table 1. We were able to obtain, by SM RT-PCR, semi-quantitative data on the expression of most of the homeobox genes even though their mRNA levels were low in some cases. In addition to genes with lost/diminished gene expression, genes with enhanced gene expression were also identified.

**Table 1:** Homeobox genes that exhibited gene expression alterations in breast and prostate cancer cell lines by SM RT-PCR.

**Both in breast and prostate cancer cells**		
UP		
	HLXB9 (MNX1)	All 5 breast and 3 prostate cancer cell lines
	BAPX1 (NKX3-2), GBX2, LHX2	7 cancer cell lines
	IPF1	6
	PAX6	5
DOWN		
	LASS3, NP_775756 (TSHZ2)	8
	CXorf43 (HDX), IRX1, POU3F1	7
	HOP (HOPX)	6
	C10orf48 (MKX), CRX, EVX2, MEOX1, PAX2, PAX8, PEPP2 (RHOXF2), VENTX2 (VENTX)	5
	CDX1, HOXA10, IRX4, NANOG, PEPP1 (RHOXF1), PROX1, PRRX1	4
	HOXA9, MEOX2	3
	IRX2, LMX1A	2
**Only in breast**		
DOWN		
	RAX	4 out of 5 cell lines
**Only in prostate**		
DOWN		
	CUTL2 (CUX2), DLX5, EMX2, HOXD10, HOXD11, POU2F3	3 out of 3 cell lines
	HOXD1, HOXD9	2

We also carried out a genome-wide gene expression analysis in normal and cancer cells of breast and prostate by DNA microarray hybridization, using Illumina's Sentrix Human-6 Expression BeadChips. Data were used to sort out the genes by a function of the frequency of the cell lines that exhibited an increased or decreased gene expression. There were 73 genes and EST sequences with enhanced gene expression in all the 5 breast and 3 prostate cancer cell lines when compared with the corresponding normal epithelial cells. Among them, 37 showed a significant increase in all cancer cell lines; these genes/EST sequences are listed in Table 2. Because cancer cell line cells multiply much more rapidly than normal cells, this list includes genes encoding for centromere proteins, kinesin and kinetochore proteins, chromatin proteins, cyclins and cell division cycle associated proteins, and enzymes involved in DNA replication and nucleotide metabolism. The list also includes v-myb myeloblastosis viral oncogene homolog-like 2. Conversely, there were 67 genes and EST sequences with diminished gene expression in all cancer cell lines examined. Among them, 13, 9, and 10 showed a significant decrease in 8 (all), 7, and 6 cancer cell lines, respectively. As opposed to the upregulated genes, those downregulated genes varied more widely, ranging from alpha-synuclein, a zinc finger protein, a matrix metalloproteinase, and amylases, to dystrophin. The list also includes genes for tumor protein p63, kallikrein-11, and cytokeratin-14.

**Table 2:** Genes that exhibited gene expression alterations in breast and prostate cancer cell lines by DNA microarray hybridization.

**UP**		
	CNPM (C22orf18), CDCA3, RAD51AP1 (PIR51), EXO1, SPC24 (Spc24), NCAPH (BRRN1), MYBL2, E2F2, CDCA5, HELLS, TTK, CDCA2, RRM2, SNG1 (SYNGR1), FLJ13909 (C16orf59), MCM10, ASF1B, CDCA2, POLE2, hmm18735 (ERCC6L), CANP (FAM111B), ORC1L, dJ383J4.3 (CENPL), CDC25C, FLJ23311 (E2F8), PIF1, CDKN2C, C13orf3 (SKA3), TRIP (TRAIP), BCL2L12, MGC2603 (C1orf135), KIFC1, FLJ13848 (NAA40), FLJ12973 (WDR76), RAD51, FLJ10520 (RFWD3), Hs.509236 (GNB2L1)	All the 5 breast and 3 prostate cancer cell lines
**DOWN**		
	ZF537 (TSHZ3), MMP28, AMY2A, DMD, TP73L (TP63), AMY2B, EDNRA, LOC163782 (KANK4), CALML3, SNCA, SERPINF1, CAPN3, ALOX15B	All the 5 breast and 3 prostate cancer cell lines
	DFZP586H2123 (PAMR1), DOC1 (FILIP1L), PTGS1, PCSK5, FLRT2, KRT14, CSTA, CSPG2 (VCAN), P2RY5 (LPAR6)	7 cancer cell lines
	FJ23221 (C1orf54), TRIM22, DLL1, KIAA0450 (PLCH2), KCTD12, KLK11, DKK3, PTGS2, Hs.16515 (COBLL1), CCND2	6 cancer cell lines

**Figure 2:**
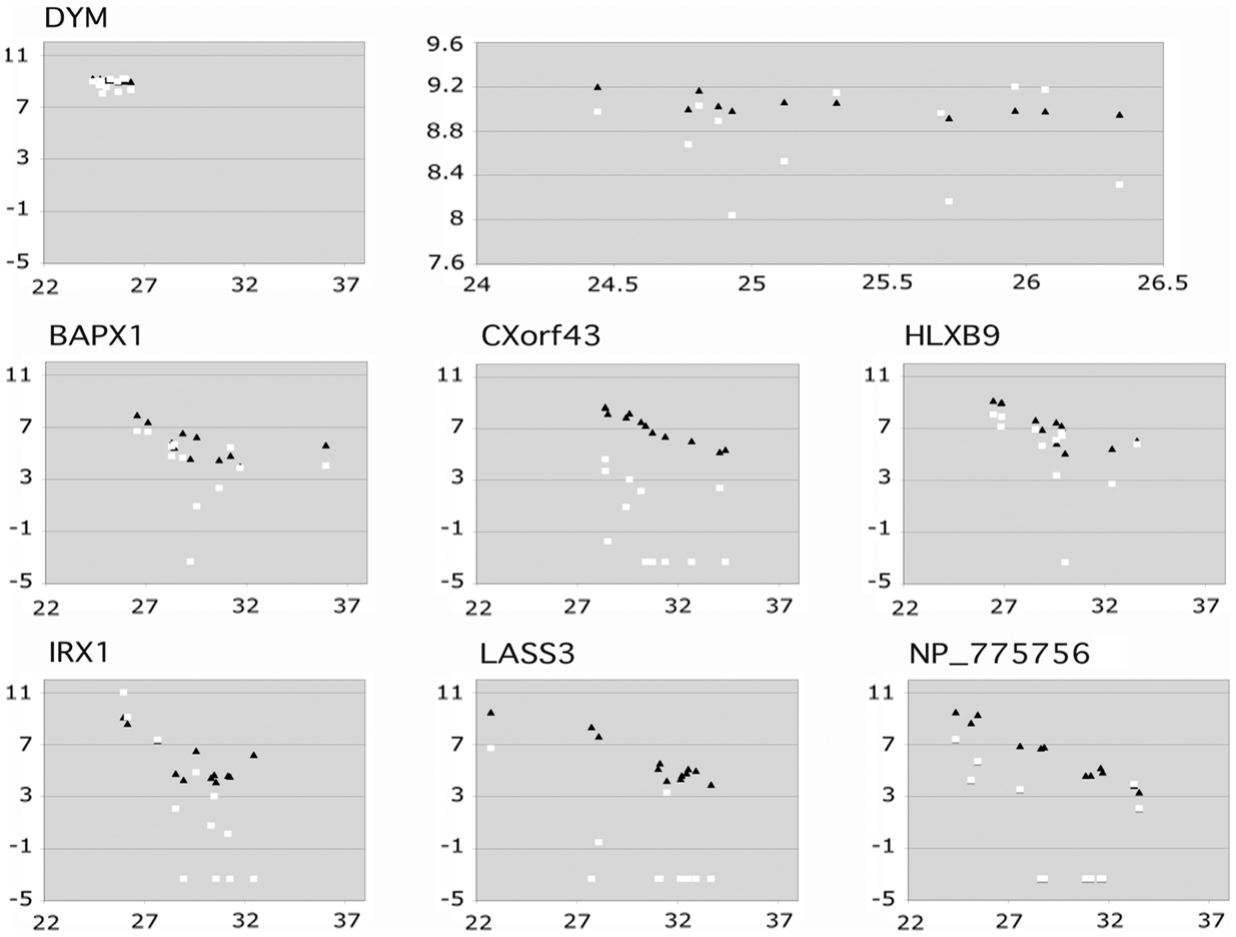
Correlation between the band intensity observed from the SM RT-PCR or the fluorescence intensity from DNA microarray hybridization and the C_t_ values obtained from the *real-time* qRT-PCR experiments. The log_2_ values of the band intensity (closed triangles) or fluorescence intensity (open squares) were plotted along the Y-axis against the C_t_ values on the X-axis. The DYM gene was used as a control. Negative and zero values obtained by microarray hybridization experiments were assigned the value of 0.1 for these graphs. The results for DYM were enlarged and are shown of the right graph on the top row. A higher degree of linearity was observed between the results of SM RT-PCR and *real-time* qRT-PCR than between the results of DNA microarray hybridization and *real-time* qRT-PCR.

**Figure 3:**
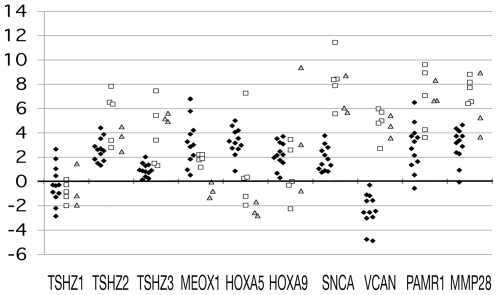
Relative expression levels of the selected genes in normal epithelial cells vs. cancer cell line cells and matched normal vs. cancer breast tissues. The expression of two-dozen candidate genes selected from the SM RT-PCR and DNA microarray hybridization screenings was determined in clinical samples. Only the representative results are shown. RNA from twelve matched pairs of normal and tumor tissues of breast was analyzed, in addition to the normal breast and prostate epithelial cells and 5 breast and 3 prostate cancer cell lines. *Real-time* qRT-PCR was employed. The expression of the DYM gene was used to normalize the expression data. The differences between the normalized C_t_ values (minus DYM C_t_) of cancer cells/tissues and those of normal cell/tissues were calculated and plotted. The black diamonds, open squares, and grey triangles represent the results of clinical cases, breast cancer cell lines, and prostate cancer cell lines, respectively. The dots above the y = 0 line indicate downregulation in tumor, whereas dots below indicate upregulation.

We performed *real-time* qRT-PCR using the same cDNA set. Two dozens of promising candidate genes from the homeobox SM RT-PCR screening were initially examined. These included BAPX1 (NKX3-2), GBX2, HLXB9 (MNX1), LHX2 upregulated genes and LASS3, NP_775756, CXorf43 (HDX), IRX1, POU3F1, and RAX downregulated genes. As a control, we also examined the expression of the DYM gene. This gene encodes Dymeclin (Dyggve-Melchior-Clausen syndrome protein) [30] and both SM RT-PCR and DNA microarray hybridization experiments showed an abundant, ubiquitous expression in all the cells and tissues examined [31]. C_t_ values were obtained for individual reactions from the r*eal-time* qRT-PCR data. We also measured the intensity of the SM RT-PCR bands using ImageQuant software and calculated the log_2_ values. Similarly, we extracted the fluorescence intensity of the corresponding genes from the DNA microarray hybridization experiments and calculated the log_2_ values. We then plotted those values against the C_t_ values for comparison. Representative results for BAPX1 (NKX3-2), CXorf43 (HDX), HLXB9 (MNX1), IRX1, LASS3, and NP_775756 are shown in Figure 2. The result for the DYM gene is also shown on the top row. The differences in gene expression observed by SM RT-PCR were confirmed by *real-time* qRT-PCR, although some of them were not detected by DNA microarray hybridization. Figure 2 also clearly demonstrates a higher degree of linearity between the SM RT-PCR and *real-time* qRT-PCR results than between the DNA microarray hybridization and *real-time* qRT-PCR. This is reasonable because both SM RT-PCR and *real-time* qRT-PCR are PCR-based techniques and the same pairs of primers that were proven useful in the SM RT-PCR were used in the *real-time* qRT-PCR experiments. As a next step, we performed *real-time* qRT-PCR using cDNA prepared from clinical specimens of breast cancer. Cancer cell lines provide a useful starting point for the discovery and functional analysis of genes involved in cancer. Alterations found in cancer cell lines, however, may not necessarily be present in the original tumors. Those changes may have been acquired during a long *in vitro* cultivation. Therefore, it was necessary to evaluate if the same differences were also observed in clinical cancer specimens. We did this, using cDNA prepared from 12 matched normal and tumor pairs of breast tissues. Together with the two-dozen homeobox genes including those ten described above, we also selected two-dozen genes that exhibited frequent downregulation in the DNA microarray hybridization experiments and determined their gene expression in the clinical breast cancer specimens. The selected genes included: ZNF537, AMY2A/2B (2 genes analyzed simultaneously), TP73L (TP63), CALML3, KRT14, PCSK5, CSTA, CSPG2 (VCAN), DKFZP586H2123 (PAMR1) and FLRT2. The DYM gene was used as a control to normalize the expression levels. The differences between the normalized C_t_ values (minus DYM C_t_) of the matched normal and tumor pairs of breast tissues were plotted. The differences between normal epithelial cells and established cancer cell lines were also plotted. Several of the genes exhibiting interesting gene expression patterns (MEOX1, HOXA5, HOXA9, SNCA, VCAN, PAMR1, and MMP28 genes) are shown in Figure 3.

*Real-time* qRT-PCR confirmed that all of the above-mentioned gene expression differences in cancer cell lines were real. However, only a subset of genes remained as candidates after performing *real-time* qRT-PCR of breast cancer clinical specimens. The results also showed that the NP_775756 and ZNF537 genes were the most promising candidates among those examined in the homeobox gene and other gene categories, respectively, because their decreased expression in tumors was observed at the highest frequency in breast cancer cases. When we searched for information on those genes using the more recent version of the Ensembl Human Genome Database, we were astounded to discover that they were renamed as TSHZ2 and TSHZ3 and categorized into the same 3-member gene family named Teashirt (*tsh*). These genes encode for proteins with widely spaced zinc finger motifs. The normal vs. cancer comparative expression levels of these genes are also shown in Figure 3, together with the expression level of the TSHZ1 gene.

While our project was underway, papers were published reporting changes in DNA methylation of the HOX A Gene Complex associated with breast cancer [32], [33]. Novak *et al.* also observed concomitant epigenetic silencing of the genes in the HOX A Gene Complex. Because we only observed the downregulation of HOXA9 and HOXA10 genes in this complex by expression analysis of breast and prostate cancer cell lines, we decided to examine, this time by *real-time* qRT-PCR, changes in expression of several other HOXA and other additional homeobox genes in clinical cases of breast cancer. The results are summarized in Table 3. We were able to confirm that the expression of many HOXA genes was lower in tumor tissues than in the neighboring normal tissues. However, the expression of those genes is not downregulated in a majority of breast/prostate cancer cell line cells, compared to the expression levels of primary culture of normal epithelial cells and/or normal tissues. In addition to the HOXA genes, we also observed discrepancies between cancer cell lines and clinical specimens in the expression of several homeobox genes that were analyzed. Among the genes found to be upregulated in cancer cell lines, GBX2 and IPF1 did not exhibit such tendency among clinical breast cancer cases. Among the downregulated genes in cancer cell lines, the correlation was weak with POU3F1 and CXorf43 (HDX), and a reverse tendency of upregulation was observed with CRX, HOP (HOPX), and IRX4. Heterogeneity of cell population among different specimens, altered expression during the *in vitro* cultivation, or else, may be responsible for the differences. Although the causes remain to be determined, we decided to focus our attention on TSHZ2 and TSHZ3, the Teashirt family of homeobox genes that exhibited the same inclination towards a diminished expression in both established cancer cell lines and clinical specimens with the highest frequency of all candidates.

**Table 3:** Expression analysis of homeobox genes in clinical specimens of breast cancer by *real-time* qRT-PCR.

Gene	Value				Gene	Value			
Name	>1	0< <1	−1< <0	<−1	Name	>1	0< <1	−1< <0	<−1
HOXA5	11	1	0	0	HOXA3	10	2	0	0
HOXA7	10	2	0	0	HOXA9	10	2	0	0
MEOX1	10	2	0	0	HOXA10	7	3	2	0
HOXA11	6	4	0	1	PEPP1	9	1	1	1
PEPP2	6	3	1	2	IPF1	6	2	1	3
C10orf48	5	3	1	3	NP_116142	6	1	0	5
LASS3	4	3	2	3	NANOG	4	3	2	3
GBX2	5	1	3	3	POU3F1	5	1	2	4
CXorf43	2	4	2	4	CRX	5	0	3	4
BAPX1	2	3	1	6	LHX2	2	2	4	4
HOP	2	0	4	6	IRX4	1	1	3	7
HLXB9	1	0	1	7					

The twelve matched normal and tumor tissue pairs of breast cancer were categorized by their subtractive values (normalized C_t_ values of cancer tissue – normalized C_t_ values of the corresponding normal tissue). The positive and negative values represent downregulation and upregulation in cancer tissues, respectively.

**Figure 4:**
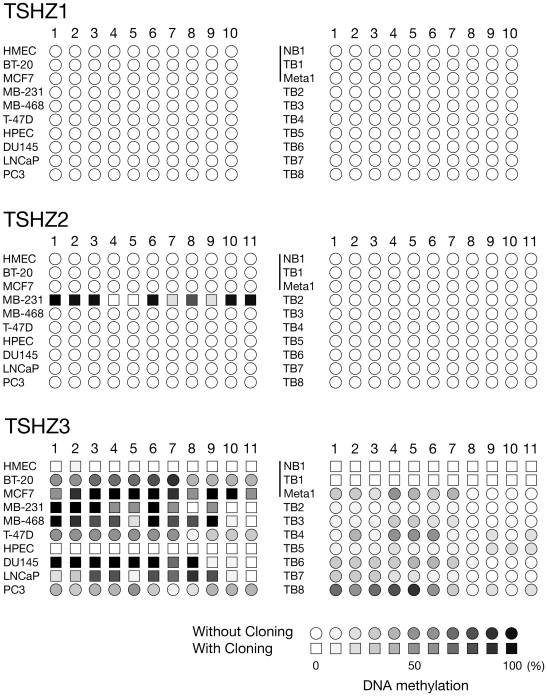
DNA methylation status of TSHZ 1, 2, and 3 gene promoter regions in normal and cancer breast/prostate cells and tissues. The DNA methylation states of individual cytosine residues from CpG dinucleotides in the amplified DNA fragments are represented schematically. The degree of methylation is indicated by increasing darkness with open and closed symbols correspond to unmethylated and fully methylated cytosines. The squares and circles indicate the results obtained by nucleotide sequencing with and without cloning, respectively.

We examined the DNA methylation status of TSHZ genes in their promoter regions in normal and cancer cells from breast and prostate tissues. The sodium bisulfite modification method was utilized followed by PCR. The nucleotide sequences of the amplified DNA fragments were directly determined without cloning. For the majority of samples that exhibited some methylation, we also determined the nucleotide sequences of the amplified fragments after cloning into a plasmid vector in order to evaluate the degree of methylation more accurately. Ten independent clones were sequenced for each sample, and the frequency of methylation was calculated. Results are schematically shown in Figure 4. Individual cytosine residues from CpG dinucleotides in the amplified DNA fragments are represented by circles (as determined by direct DNA sequencing without cloning) or by squares (as determined by cloning). The percentages of methylated cytosines are indicated in grey scale (open circle/square <10% methylation, closed circle/square >90% methylation). The promoter region of the TSHZ1 gene, which is expressed in both normal and cancer cells/tissues of breast and prostate, was found unmethylated irrespectively of the normal/cancer status (top panel). The TSHZ2 gene promoter region was also unmethylated in all the cells and tissues examined with the exception of the MDA-MB-231 breast cancer cell line cells (middle panel). The methylation status of the TSHZ3 gene promoter depended on the pathological state of the cells (bottom panel). It was unmethylated in normal epithelial cells, as well as normal tissues, of breast and prostate, whereas in all the cancer cell lines examined the corresponding region was partially or entirely methylated. We also examined 7 additional breast tumor tissues, and found that several had some degree of methylation at the TSHZ3 gene promoter region analyzed.

## Discussion

Cancer is the result of a series of genetic and epigenetic mishaps subjected to natural selection. All cancers involve a disruption of normal restraints in cell proliferation, differentiation, and survival. Two major routes exist that contribute to uncontrolled cell proliferation; activation of proto-oncogenes and inactivation of tumor suppressor genes [34]. Structural changes in critical proteins caused by gene mutations may either activate or inactivate their function. The activation may also occur through an increased gene copy number and enhanced gene expression whereas the inactivation may also occur by decreasing the copy number and downregulation of gene expression. Therefore, changes in copy number and expression have been used as landmarks to identify dozens of cancer-related genes. In this work we searched for genes that exhibit consistent differences in expression between normal epithelial cells and established cancer cell line cells. We analyzed breast and prostate cancers, which progress from an early, sex hormone-dependent, organ-confined disease to a highly invasive, hormone-independent, metastatic disease that invades regional lymph nodes and distant organs, such as the skeletal system. Although they are similar, breast and prostate cancers arise in two different systems. Therefore, we aimed to identify genes that exhibited altered expression in breast, prostate, or both cancers. We also decided to use normal and cancer cells rather than tissues to circumvent the heterogeneity and contamination problems of tissues. Additionally, we took two different approaches: SM RT-PCR and DNA microarray hybridization. The former was targeted to homeobox genes while the latter was used for the genome-wide analysis.

The search resulted in the identification of dozens of genes that exhibited altered gene expression in breast/prostate cancer. Many of the HOXA genes were downregulated in clinical specimens of breast cancer. However, they were excluded from the candidate list because several breast/prostate cancer cell line cells did not satisfy the criteria when compared with the expression levels of primary cultures of normal epithelial cells and normal tissues. This implicates that different selection criteria would have resulted in different results. Amazingly, after *real-time* qRT-PCR screening using clinical specimens of breast cancer the top candidates from the two separate approaches turned out to belong to the same Teashirt family of genes. When we constructed the SM RT-PCR system of homeobox genes, ZNF537 (TSHZ3) was not annotated. The NP_775756 (TSHZ2) gene was not listed among the top candidates from the DNA microarray hybridization approach because its expression was lower in the primary cultures of normal epithelial cells and several dozens of better candidates were identified. In fact we might have missed those interesting homeobox genes if we had not performed both the SM RT-PCR and DNA microarray hybridization experiments. Because the TSHZ2 gene was selected from homeobox genes and the TSHZ3 gene was selected from the sum of 46,000 genes/ESTs and as there are only 3 members in the Teashirt gene family, we have concluded that our finding is not a mere coincidence of one in 3 millions (1/194×1/46,000×3 = 1/2,974,666). A decrease in expression of the TSHZ2 and TSHZ3 genes was observed in 100% of the breast cancer clinical cases examined. The downregulation of these genes may also be observed in several other types of cancers, very probably including prostate cancer. In comparison, the TP53 gene (p53) is altered in 40% of breast carcinomas cases (Catalogue of Somatic Mutations in Cancer (COSMIC) database: http://www.sanger.ac.uk/genetics/CGP/cosmic/), and although germline alterations in the BRCA1 and BRCA2 genes are involved in many cases of hereditary breast and ovarian cancers, the hereditary form of these diseases account for only 5 to 10% of the total cases.

Because the downregulation of mRNA stationary levels is likely caused by decreased transcriptional activity, it is necessary to examine the alterations in chromatin structure surrounding the TSHZ2 and TSHZ3 gene promoters. As an initial step, we examined changes in their DNA methylation. All three TSHZ genes contain regions highly rich in CpG dinucleotides around exon 1, suggesting that those regions represent CpG islands. Therefore, we anticipated that the TSHZ2 and TSHZ3 promoters might be hypermethylated in the non-expressor cancer cell line cells whereas in the expressors they would be hypomethylated, as it has been demonstrated with other tumor suppressor genes [27], [28]. We also expected no difference in the methylation status of the TSHZ1 gene in the TSHZ2 and TSHZ3 expressor and non-expressor cells/tissues. The DNA methylation analyses showed that the promoter of the TSHZ1 gene is, as we expected, unmethylated in all the examined cells and tissues. Furthermore, the methylation status of the TSHZ3 gene promoter correlated well with its gene expression (unmethylated in expressor cells/tissues and methylated in non-expressor cells/tissues) as we also anticipated. On the contrary, the results of TSHZ2 gene promoter were somewhat unexpected. It was found unmethylated except in the MDA-MB-231 cells, suggesting the presence of other gene silencing mechanisms than DNA methylation. One possible mechanism may be a diminished or abolished expression of an upstream transcription factor(s) responsible for the TSHZ2 gene expression. In *Drosophila*, the Teashirt (*tsh*) gene is required for the development of embryonic trunk segments [35]. Additionally, *tsh* is also necessary for midgut morphogenesis, the patterning of adult eyes, and the development of the proximal portion of adult appendages [36], [37], [38]. In mice, TSHZ1 regulates posterior identity in brain and cranial neural crest cells [39], and is required for axial skeleton, soft palate and middle ear development [40]. The three mouse Teashirt genes could rescue both the homeotic and the segment polarity phenotypes of a *tsh* null fruitfly mutant [41]. The mammalian genes are also expressed in domains both dorsoventrally and rostrocaudally restricted, with major changes in expression levels coinciding with compartment boundaries [42]. Mice that are null mutant for TSHZ3 exhibit congenital pelvi-ureteric junction obstruction with defective smooth muscle differentiation and absent peristalsis in the proximal ureter, suggesting a role in organ development [43], [44]. Recent reports have correlated the TSHZ genes to human diseases: reduced expression of TSHZ3 protein to Alzheimer disease [45] and deletion of the TSHZ1 gene, which is located at the 18q22.3 critical region, to 18q deletion syndrome [46]. Patients with the latter syndrome display a multiple-anomaly disorder associated with mental retardation, white matter anomalies in the brain, growth hormone deficiency, congenital aural atresia, orofacial cleft, and palate abnormalities.

Apparently Teashirt genes/proteins have never been associated to carcinogenesis except that TSHZ1 protein was found reactive with an autologous IgG from patients with colon cancer (NY-CO-33 colon cancer antigen) [47]. Therefore, it will be interesting and necessary to determine the role and significance of THSZ2 and TSHZ3 transcriptional inactivation in cancer. We hypothesize that their gene silencing may play an active role in carcinogenesis. There already exists some evidence to support this. The Teashirt proteins were found in the Wnt signaling pathway in *Drosophila* and, in humans, as part of a gene-silencing complex in neuronal cells. In *Drosophila*, the Wnt protein Wingless acts to stabilize Armadillo inside cells where it binds to at least two DNA-binding factors, which regulate specific target genes. One of the Armadillo-binding proteins is the Teashirt protein [48]. Upon an extracellular signal (e.g. Wg/Wnt), Arm/β-catenin seems to recruit the Teashirt protein and stimulates the entry into the nucleus, where the bipartite complex can collaborate with general DNA-binding factors to regulate specific target genes of the pathway. In humans the activation of gene transcription by β-catenin plays a critical part in carcinogenesis, and actually, three regulatory genes in the Wnt signaling pathway are found to be mutated in primary cancers [49]. The study on human neuronal cells revealed another role of Teashirt proteins as transcriptional repressors [45]. The FE65 adaptor protein, which can bind to the amyloid protein precursor, simultaneously recruits SET, a component of the acetyl transferase inhibitor, and the Teashirt protein, which in turn recruits histone deacetylases, to produce a gene-silencing complex [45]. Interestingly, decreasing expression of Teashirt (due to genetic or other causes) and increasing expression of caspase-4, a target of the silencing complex, were correlated with progressive cognitive decline in AD patients.

We do not know if the downregulation of TSHZ2 and TSHZ3 genes is the result of shutting down gene expression when normal expressor cells dedifferentiate and become malignant or if it is the result of transcriptional activation when the non-expressor stem cells, which develop into cancer cells, differentiate into epithelial cells. Regardless, it is evident that the difference in gene expression allows discrimination of normal, differentiated epithelial cells from undifferentiated (dedifferentiated) cancer cells, and therefore, it is a potentially useful diagnostic cancer marker. We do not know whether Teashirt proteins work as activator or suppressor of transcription in epithelial cells, either. However, it is likely that decreased TSHZ gene expression not only results in the decline of the TSHZ proteins but it also elicits multi-faceted changes in the expression of downstream genes. The CASP4 gene may not be a target gene in the epithelial cell system. Rather, one or several of such genes that are shown to be either upregulated or downregulated in cancer cells in Table 2 may turn out to be the target genes. Secondary changes in the protein profile may provide useful targets for pharmacologically active compounds. In addition to elucidate the downstream genes, there are many additional questions regarding the functions of the Teashirt proteins in carcinogenesis. We hope to provide definitive answers in future studies.

## Materials and Methods

### Systematic Multiplex RT-PCR (SM RT-PCR)

To measure the expression of 194 homeobox and related genes we used the following total RNA samples: a normal and a primary breast tumor tissue from a patient with invasive ductal carcinoma, a normal and a primary carcinoma tissue of prostate from a patient with prostate cancer, another normal prostate tissue, and a hyperplastic prostate tissue, primary cultures of normal mammary and prostate epithelial cells, and 5 mammary (BT-20, MCF7, MDA-MB-231, MDA-MB-468, and T-47D) and 3 prostate (DU145, LNCaP, and PC3) cancer cell lines. The normal and cancerous human tissues were obtained from the Cooperative Human Tissue Network (CHTN). The primary cultures of human epithelial cells and the established cancer cell line cells were purchased from Cambrex and American Type Culture Collection (ATCC), respectively. We prepared cDNA by reverse-transcription using oligo dT primers and the Advantage RT-for-PCR Kit (BD Biosciences-Clontech). We followed the SM RT-PCR experimental protocols described previously [29], [50], [51], [52]. Complementary DNA samples were used as templates to examine gene expression. Small aliquots of the SM RT-PCR reaction products were loaded onto 8% polyacrylamide gels and electrophoresed. The gels were stained with ethidium bromide and TIFF-formatted pictures were taken.

### DNA microarray hybridization

In order to determine the genome-wide gene expression, the same cDNA preparations that were used for SM RT-PCR were also employed in microarray hybridization experiments. The samples analyzed were a normal breast tissue, a normal prostate tissue, primary cultures of normal mammary and prostate epithelial cells, and 5 mammary and 3 prostate cancer cell lines. Illumina's Sentrix Human-6 Expression BeadChips, which contained probes from the entire 23,000 RefSeq collection and an additional 23,000 other expressed sequences, were used. We followed Illumina's protocol to prepare biotinylated cRNA and we hybridized with the BeadChips. Fluorescence intensity was measured with Illumina's BeadStation 500. Raw data were generated and then normalized using the Beadscan 3.0 software.

### Real-time qRT-PCR

To measure the expression of selected genes two sets of cDNA were analyzed: the same set of cDNA from the cells and tissues that were used during the DNA microarray hybridization experiments and another set from 12 matched pairs of normal and tumor breast tissues. The reagent was the Power SYBR Green PCR Master Mix (Applied Biosystems) and the primer pairs were the same used in the SM RT-PCR experiments. The PCR products yields were monitored using the Mx3000p system (Stratagene) under default conditions, with the exception of an increase in the annealing temperature to 60°C instead of 55°C. Data were analyzed using the MxPro software. Cycle threshold (C_t_) values were obtained for each individual reaction, and the C_t_ of the ubiquitously expressed DYM gene was subtracted to obtain the normalized values.

### DNA methylation analysis

DNA methylation status was determined for TSHZ1, 2, and 3 gene promoters, using genomic DNA from the same cells/tissues analyzed for gene expression. We employed the sodium bisulfite modification method followed by PCR and DNA sequencing as previously described [53]. Briefly, DNA sequences surrounding the transcription initiation sites of the TSHZ genes were retrieved from the Ensembl Human Genome Database, and the CpG-rich regions were identified. Genomic DNA was treated with sodium bisulfite under the conditions that allowed the conversion of cytosine, but not 5-methylcytosine, to uracil [54]. The modified DNA was treated with sodium hydroxide followed by ethanol precipitation. DNA fragments containing multiple CpG dinucleotides from the TSHZ gene promoters were PCR-amplified and directly sequenced, using primers corresponding to the bisulfite-converted sequences without CpG dinucleotides. The nucleotide sequences of the primers used are:TSHZ1-F(GGGAGGAAAAGGATAGTTTGTAT), TSHZ1-R (CAACTTTCTCTCCCCCTCTCTCCT), TSHZ2-F (GGAGGAGTTTGTTAATGTTTAG),TSHZ2-R(AAAATCTAAAATTCACTCACTCACAC),TSHZ3-F (GGGGGATTGTTTGGTGTT), and TSHZ3-R (CATCTAACAATACCCAAACCCTAT). The locations in the human genome of the amplified DNA fragments are: TSHZ1 (Chr:18, 72923259-72923378), TSHZ2 (Chr:20, 51588877-51589110), and TSHZ3 (Chr:19, 31839537-31839698) (Ensembl release 59-Aug 2010). The DNA methylation status at individual CpG sites was manually annotated. For several specimens, the PCR reaction products were also cloned into the pCR2.1 plasmid vector, using the T-A cloning method. After DNA transformation of competent *Escherichia coli* bacteria, plasmid DNA was prepared from independent clones, and ten clones were sequenced in order to obtain more accurate estimates of methylation frequency.

## Supporting Information

Table S1 The list of the genes analyzed by SM RT-PCR is shown, together with the nucleotide sequences of primers, their concentrations used, and the sizes of the amplified DNA fragments.
(PDF)
