# Supplementary material for: Rare and Frequent Promoter Methylation, Respectively, of TSHZ2 and 3 Genes That Are Both Downregulated in Expression in Breast and Prostate Cancers
Source: PLoS One. 2011 Mar 14;6(3):e17149. doi: 10.1371/journal.pone.0017149 (PMC3056709; doi:10.1371/journal.pone.0017149)
Supplement: Table S1 — The list of the genes analyzed by SM RT-PCR is shown, together with the nucleotide sequences of primers, their concentrations used, and the sizes of the amplified DNA fragments. (PDF) [file pone.0017149.s001.pdf]

Set	Gene Name	Primer 1 Sequence	Primer 2 Sequence	Fragment Size (bp)	Primer Concn (nM)
1					
	**HOXA6**	GAGCGCCAGATCAAGATCTGGTT	CTCCCCTGAAGCTGCGGAAGCCCC	240	45
	**HOXA9**	GAGAGGCAGGTCAAGATCTGGTT	GAGGTTTAGAGCCGCTTTGTGCG	217	45
	**HOXA7**	GAGCGCCAGATTAAGATCTGGTT	GGCCCCGGATCGGCCCCTCATTC	200	227
	**HOXA2**	GAGAGACAAGTGAAAGTGTGGTT	AGAAGGGCCCCAGAGACGCTAAG	182	91
	**HOXA5**	TTGCCTCTCCGAGAGACAAATTAAAATCTGGTT	ACTGACACTACGCGGGATCCGCTAATA	173	159
	**HOXA10**	ACGGACAGACAAGTGAAAATCTGGTT	GGAAGTGAAAAAACCGCGTCGCCTGG	141	45
	**HOXA13**	GAGCGGCAGGTCACAATCTGGTT	TTCTGAAGCGTTTCTTCAAGTTGCCTTCTTGC	128	45
	**HOXA4**	GAGCGCCAGGTCAAGATCTGGTT	CTTTCCCTGGTGGGCCGGCAGAGGC	124	45
	**HOXA1**	GAGACCCAAGTGAAGATCTGGTT	CCTTCTCGTCGTTTCCTGGCG	112	45
	**HOXA3**	GAGCGCCAGATCAAGATCTGGTT	CTTGGAGACTGGCCCCCCGAT	101	45
	**HOXA11**	GATCGTCAAGTCAAAATCTGGTT	TGGATTTGCTGAGTAGTACTGTAAACG	90	159
2					
	**HOXB3**	GAGCGGCAGATCAAGATCTGGTT	GGTAGTTGGAGGGCAGCGCGTAG	241	75
	**HOXB5**	GAGCGCCAGATCAAGATCTGGTT	GCAGGCTTGTGGGAACCGGTC	220	50
	**HOXB1**	GAAACACAGGTCAAGATTTGGTT	GGAAGCCCCATTGGTGGCTAGGT	205	50
	**HOXB7**	GAAAGACAGATCAAGATTTGGTT	CTGGGCTTCTCTTCGTCTCCCTTTCTCATG	180	175
	**HOXB8**	GAGAGACAGGTCAAAATCTGGTT	CTTGTCGCCCTTCTGCGCGTC	168	250
	**HOXB9**	GAGAGACAAGTCAAAATCTGGTT	GCACTGGCTTTGCAGTCGTCACAT	156	50
	**HOXB4**	GAGCGCCAGATCAAGATCTGGTT	CGCGGGGGCCTCCATTGGGC	142	100
	**HOXB13**	GAGCGCCAGATTACCATCTGGTT	CCAGGACACCCCCACTTTCGC	134	50
	**HOXB6**	GAGAGGCAGATCAAGATATGGTT	CCAGCACCTTACTCGGCCTGTTTTTCTTCC	125	50
	**HOXB2**	GAAAGGCAGGTCAAAGTCTGGTT	GGTCGCAGATGTCCTCCAGGG	121	250
3					
	**HOXC13**	GAGCGCCAGGTAACCATCTGGTT	GCGCGGTTCTTCTCTTTCCTTAACA	215	56
	**HOXC6**	GAGCGACAGATCAAAATCTGGTT	GGGAGAGAGGGGTGGCAGGGAC	195	111
	**HOXC8**	GAGAGACAAGTGAAGATCTGGTT	GGGGTCTTTCTTTTTTGCTTAGTCCTTGTT	180	194
	**HOXC4**	GAGAGGCAGATCAAAATCTGGTT	GTGGTCTTCAGAAGTACCCGGGGTA	150	83
	**HOXC12**	GACCAGCAGGTCAAGATCTGGTT	TTTGCTCTCTGCCAGGGACAGG	142	111
	**HOXC9**	GAGCGGCAGGTCAAAATCTGGTT	TTTCCCTTGGCTGTGCTGAGG	126	56
	**HOXC11**	GACCGACAAGTGAAAATTTGGTT	AAAGGGCCCGGTCTGCAG	118	556
	**HOXC10**	GACAGACAAGTCAAAATCTGGTT	CGCTCTCAGGTGAAATTAAAATTGGAGGT	110	278
	**HOXC5**	GAGAGACAGATCAAGATCTGGTT	CCCCGCTGCCTCTAAAGAGCCT	95	194
4					
	**HOXD4**	GAGCGCCAGATCAAGATCTGGTT	CCAGGGTCCCCACTTCTATAAGGTCG	201	83
	**HOXD9**	GAGAGACAGGTCAAAATCTGGTT	CTGGAGAGTTTCTGGAAATCAAGCACCAAACA	185	278
	**HOXD11**	GACCGGCAAGTCAAAATCTGGTT	GAGCAGGCTGGTGGGAAGG	162	194
	**HOXD8**	GAGAGACAGGTAAAAATCTGGTT	CTGTCTTCCTCCAGCTCTTGGGC	140	278
	**HOXD13**	GAGAGACAAGTGACCATTTGGTT	GACAACCGAATGGCTTCTAAGCTGTC	130	111
	**HOXD10**	GACAGGCAGGTCAAGATTTGGTT	TCAGACCGGCCTCAGACCTAAGA	122	111
	**HOXD1**	GACACGCAAGTCAAAATCTGGTT	GAAGTTGGAGGGGAGCCACAG	106	111
	**HOXD12**	GACCAGCAAGTCAAAATCTGGTT	CACGCGCGCGGCTAGTAG	98	194
	**HOXD3**	GAACGCCAGATCAAGATCTGGTT	ACTGGCTAGCCGGCGAGTGCAGGATG	94	278
5					
	**GBX1**	ACAGCATTTACCAGCGAGCAGCTT	CCAGAACGGCTGCTCACATTGC	191	46
	**BARHL1**	AGATCTCCAGCTCCAGGGACAGT	TGCGTGTCGGTGAGGTTGAGCGA	175	52
	**MSX1**	AAACACAAGACGAACCGTAAGCCG	GGAACCATATCTTCACCTGCGTCT	160	202
	**HLXB9**	CGCTCTCCTACTCGTACCCGCA	GAAGTCGGGCATCTTAGGCAGGAT	146	433
	**NP_001009812**	GTGTCGAGGAGGGCGTCGGAGTTT	GCGGCAGGCCTGTGAGTTGTTT	133	29
	**GBX2**	GCTGATGATGATGCAGCGCCCG	GGTAGCCGGTGTAGACGAAATGG	110	577
	**DLX3**	CATGGTGAATGGGAAGCCCAAGAA	GTACTGGGCCTTCTGGAAGCGG	100	288
	**DLX1**	AGTGCGCTTCAATGGCAAGGGAAA	CTGGAACCTCCGGTTCAAAGCCT	91	87
	**DLX6**	AAGCCTCGGACCATTTATTCCAGC	TCTGGAAGGGCCAGATACTGTGT	83	87
	**DLX4**	TCCGCAAGCCGAGGACCATCTACT	AGGTACTGCGTGTGCTGGAAAC	76	58
	**DLX5**	AGAAGACTCAGTACCTCGCCTTGC	TGTGTTTGTGTCAATCCCAGCGAG	70	87
	**DLX2**	AGAAGGAGGACCTTGAGCCTGAAA	GGGTTTCCGGACTTTCTTTGGCTT	65	288
	**LBX1**	GAGGAGCTCGCCAGCAAGACGTTTA	CTTCGGCTGCCTGCAGAACGCT	61	346
6					
	**PAX7**	ACTCCATCAAGCCAGGAGACAG	ATAAACAAGCAGGAGGCCCTGG	193	87
	**PAX1**	CGGACGCCCTCAGTAGCTTACA	ACTGGCTTCCTCCTCCGCGAGT	177	101
	**PAX9**	TACATGACCTACAGTGCTGCTCCT	GGAAGCCGTGACAGAATGACTACCTT	162	46
	**PAX2**	AATGGAGATTCCCAGAGTGGTGTG	CTGATTTGATGTGCTCTGATGCCTGG	148	58
	**PAX3**	GGCCAGCTGCAGTCAGAGACTAGA	TACTGCCCATATTGGTAGCCTGTG	135	46

	**PAX8**	ATCCTCACTCACCCTTCGCCATAA	GAGCCGACTTGCTGCAGATCCAAA	112	101
	**PAX5**	AGACTTCCTCCGGAAGCAGATGCG	GTGAAGATGTCTGAGTAGTGCTGCCT	102	72
	**PAX4**	AACCACTGGGTCCCTCCTGCTAT	AGGGCTTGAGACAGGCTTTAGGT	93	52
	**PBX3**	GCAAGTTTCAGGAAGAAGCCAACC	TTGTTCTGCACAGCTGCTGCTACT	85	115
	**PBX4**	GCGACGGAAGTGCTGAATGAGTAT	CAGCTCTTCTTTGGCTTCTTCGCT	78	72
	**PAX6**	TCGGTGAATGGGCGGAGTTATGAT	CATTGGCTGACTGTTCATGTGTGTC	72	72
	**PBX2**	GTCTCCAACTGGTTTGGCAACAAGAG	CCTCCTCTTGGAACTTTCCGATGT	67	101
	**PBX1**	CTATTCCCATCTCAGCAACCCTTACC	CACTTCTTGGCTAACTCCTCTTTGGC	63	101
**7**					
	**NP_078843**	GCCTTGGAAACTTTGGACCGTCTT	TTGATTGGTAGTGCAAGGCTGTGG	213	35
	**CUTL1**	ACAGCTCAGTCAGTGACAGCCA	GTGGCGAGGTCTTCGATGGTTT	196	35
	**NP_775756**	CTCACCTGGGTTTCCAAATGAAGGAC	GTTTGCTCACAAATGTCCGACAGC	180	46
	**CUTL2**	GGAAAGCATCCTGGGTCTGACA	TCCAGCTTCTTCATATCCCTCAGC	165	58
	**TLX1**	CGTCCTTCACACGCCTGCAGAT	CACTTTGTCCGCCGGTTCTGGA	151	58
	**TLX3**	AGCTGCAACACGACGCCTTCCAAA	TGACAGCGGGAACCTTGGAACTAT	138	288
	**LASS2**	AAGATGAACGCAGTGACCGGGAA	TCTTACGATGGTTGTTATTGAGGATGGG	126	87
	**LASS4**	ACACCACATACTACGAGTCCATCAGC	AGAATGAGGCAAGACCAGAACACG	115	72
	**LASS1**	AGGTCCTGTATGCCACCAGTCA	AGAGGTTCATAAGGGTGAGCAGCA	105	46
	**LASS5**	AGCAGCTCAGAGGAAGAAGATGTG	TCCCATGTGACCATTCACCCGATT	96	46
	**LASS6**	AGGACTCAGAACCTCCGGGAAAGAAT	GAGCCAGTCAGGAGATACCCGTT	88	52
	**TLX2**	TTCCTGCGCCAGAAGTACCTGG	TTTGACCTGTGCGTCGGTCATGC	81	577
	**LASS3**	TGCACGCTGATCTTGCCTATGT	GATCATGAGCTGTAGGTTGAGGAAGATG	75	72
**8**					
	**HMX3**	GAGATCATTCTGGAGGAGAGCGACT	AAGACTGTGCGCGTCTTCTTCTTG	182	231
	**MSX2**	ATGGCTTCTCCGTCCAAAGGCAAT	TTGTCGGACATGAGCGCCTCCA	167	231
	**NKX2-4**	TCTTCTCGCAGGCGCAGGTCTAC	GTTTCATCTTGTACCGGTGGTTCTGG	153	404
	**MIXL1**	CGGAGATTATCCTCAACCACTGTGC	ACCTTGGGAGCTAGAGTCAGAGAT	140	115
	**IRX2**	GTGGACTCGCTCACGGATCACT	TCGTCCTCCAGGTCGTCATACTTGT	128	288
	**LHX4**	ACTTTGTCTACCACCTGCACTGCT	TCTCGTAGTCTTCCTTGCACACCA	117	58
	**ARX**	CTGCTGAAACGCAAACAGAGGC	CTGGTGAAGACGTCCGGGTAGT	107	288
	**POU6F1**	ATTGCTAAGGAGCTCAACTACGACCG	TGAAAGACGTTCAGCTTGCTGGTG	98	58
	**EN2**	CTCGGACTCGGACAGCTCGCAA	TCCGAGTAGCGCGTACAGTAGA	90	404
	**GSH1**	AAGTGCTCCGAGGATGACGACGAAT	TAGGGAGTGACCGTAAGATCCCG	83	87
	**TITF1**	AAAGCACACGACTCCGTTCTCAGT	CCTCCATGCCCACTTTCTTGTAGCTT	77	144
	**POU4F1**	TCGCCGAGAAACTGGACCTCAAA	GCTTCTGCTTCTGTCTCTGGTTGC	72	144
	**SHOX**	TGAATGCAAAGAGAAGCGCGAGGA	TCTGTTTCAGCTTGGTCTGCCCGT	68	115
**9**					
	**VSX1**	AAGATAAGTTGGCAGGACTCTGGG	GAGTTGGAGCCTCCTTGAGCAC	199	35
	**GBX2**	ACTCAGCGAGGTGCAGGTGAAA	GCCTGTTCTAGCTGCTGATGCT	183	87
	**LBX1**	GGCCTTCACCAACCACCAGATCTAT	TCCTCCAGGTCCCGCTTGAGCTTAG	168	87
	**IPF1**	TAGAGCTGGAGAAGGAGTTCCTATTCAACA	CCGCGCTTCTTGTCCTCCTCCTTT	154	87
	**NKX2-8**	GCCTACCAGCACTTAGCATCCC	ACGGAGAGCGTTCCAGGCGTTC	141	144
	**NKX3-1**	TTCAGCCATCAGAAGTACCTGTCG	GGAGAGCTGCTTTCGCTTAGTCTT	129	144
	**BAPX1**	GCTTTAACCACCAGCGCTACCTGT	CGCTTTGTCTTGTAGCGACGGTTC	118	202
	**VAX1**	CCGTTAACAATGGCTGGTTCGCTA	CCCTTCTTTATTGTTGGTCCGGGAGT	108	231
	**DLX3**	ATGGTGAATGGGAAGCCCAAGAAG	GTACTGGGCCTTCTGGAAGCGG	99	144
	**NKX2-2**	AGCACATGCAGTACAACGCCCAGTACA	CAAGTCCACTGCTGGGCCTGGAC	91	87
	**TLX1**	TTCCAGAAGAGCCTGGCACAGC	ATTCTGCAGGGCGAAGAGCGACGA	78	404
	**HMX2**	TCGACCTGAAGCGCTACCTGAGCA	AGATCTTAACCTGCGTCTCGGTGA	84	46
	**HLXB9**	CTCAACAAGTACCTGTCGCGGCCCAA	CTGGGTCTCGGTGAGCATGAGC	69	144
**10**					
	**TLX3**	GATCTTACAGTGTGAACCTGAGCCTAGC	TGTGAAGCGGTCTTTCACGAAGC	200	231
	**BARX1**	AGCAGGCGGCGGTGTTCAAGTT	TTCTTGGCTTTGGTGCCTGGCT	184	115
	**TLX2**	GATGCTGGGTCCACACAACCTCCCA	GTAGCCCGAGGCTCCGTGGTAT	169	173
	**LHX6**	AGGAGCTCTGGACAAGGACGAG	TGAGCAGATATCGGTCCAGGATCTC	155	87
	**ISL2**	AGATCCACGACCAGTTTATCCTGC	TAGGTCTTCCCGTCTCTCACGAA	130	173
	**SIX3**	CTGTTGCGGGAGTGGTACCTACAG	TGCCGCCGGTTCTTAAACCAGTTGCC	119	87
	**SHOX2**	AAATCAAGCAGAGGCGAAGTCGGA	AGTTCCTCTCGCATGAAGGCGT	109	115
	**PITX2**	CGGCCGTTGAATGTCTCTTCTCCAAA	AAGAACTTGCTGCTGGCTGCCTCTTT	100	87
	**HOP**	TGCAGATCCGTCACAGACTAAGGA	AGCTTGGTTAAGCGGAGGAGAGAA	92	72
	**NKX6-2**	ACGACGACGAATACAACCGGCCC	TTCGAGGGTTTGTGCTTCTTGAGC	85	288
	**GSCL**	TTCAAGAACCGCCGGGCCAAAT	ACTTCTTGACGCCGGGCAGGAG	79	115
	**EMX2**	AGCTGGAGGAAGAAGGCTCAGATT	GGCGATTCTCCACCGGTTAATATGGT	74	72
	**HHEX**	AAAGGAAAGGCGGCCAGGTGAGATT	TGCGTCTCGAATTTCTTCTCCAGC	70	72
**11**					

**POU3F1**	TACGGTAACGTGTTCTCGCAGAC	TCGATGGACGTGCGCTTCTTGC	191	469
**POU3F2**	AGCATAGACAAGATCGCAGCGCAA	ACCAAACTCTCACCACCTCCTTCT	175	250
**POU6F2**	GCCAAACTGAACCCTGGCCTTT	TCCCGATAAACTCGGTCAGGTTCT	160	63
**POU6F1**	TCGAGAAGCTAGACATCACACCCA	TTTGCGTTTCTTGGAGGGCTCG	146	50
**POU4F1**	TGAACAAGCCTGAGCTCTTCAACG	ATGGCGGCGATCTTCTCGGACG	133	250
**POU4F2**	GCTCACCAAGCCTGAACTCTTCAA	TTCAGAGGAGGGCCGAGGCTGAAT	121	188
**POU2F1**	TGCAACTGGGAACCTGGTATTTGC	TAACAGGGTTGCTGGTGAGCAGA	110	63
**POU3F4**	ACGCATTTCCTCAAGTGTCCCAAG	TACAGAACCAGACACGCACCACTT	100	156
**POU2F2**	CAGAGGAGATCCTGCTGATCGCCGAG	CGTTTCTCCTTCTGGCGCCGGTT	91	63
**POU1F1**	AGATGCTCTGGAGAGACACTTTGG	CCAGATTCAGTTCTTCAGCCATCCTC	83	94
**POU5F1/L/N**	TGGTCCGAGTGTGGTTCTGTAAC	TCTCGTTGTGCATAGTCGCTGCTT	70	63
**POU2F3**	AAGGAGAAGCGAATCAACTGCCCT	GGGAGTTGTAGACAGGTGGTTTGA	61	94
**12**				
**ALX3**	CTGAGGAGAAGACCTCCAAAGCTG	AATGTGCTGAAGGTCGTGCGGTTA	211	94
**VSX1**	AGGAAGTGAAGATAAGTTGGCAGGAC	AGGGTGCACTTTCTTGGTCTCCT	178	50
**CHX10**	TCCCAGGAGGAACTGAGGGAGAA	TCTCCGCCGGCCTGTCTTCATC	163	56
**ALX4**	ATTCAGAACCCGTCCTGGCTCG	ACACTCAGGAAGTCGGTGACGCT	149	125
**CART1**	TCGCCTCGGACAGATTCCAGTTA	CAAATGCATGTCCATTGGTTGCCC	124	38
**SHOX2**	TACATGATGTTCCCAGCACCGCCCTT	TTGCTGGTGGTCTTGGCGGCTG	113	219
**HESX1**	CCCAATGCCAGAAGAAAGAGCTTC	TGGTCTTCGGCCTCTATACCAACT	103	94
**LMX1A**	GAGTGCTGGGATGGAAGGAATCAT	ATCTGAGCTGTAGACACTCTGCTCG	94	78
**ISL2**	CAGTGGAGGTGCAGACGTACCA	TTGGAAGGCGGGTTGGTCCAGGT	86	78
**ISL1**	TGGCTTACAGGCTAACCCAGTGGAA	CAAGGCGAAGTCGCTCAGTACTTT	73	125
**LMX1B**	GAGGGCATGATGGCTTCCTACA	CTCTGTTCCATGGCCACGATCT	68	94
**CXorf43**	ACTGCTGTCAGATTTACCTCCTGAATTAGA	AGGCTCCAGTGAGGCATGATTGAAA	64	156
**13**				
**PEPP2**	AGGTCAAAGAGGAGGAAGAGGATGC	CTTCCCATAGGTGGCCAGGAACT	162	60
**PITX2**	ACCCGTCTAAGAAGAAGCGGCAAA	GCTTCCGTAAGGTTGGTCCACACA	148	375
**PEPP1**	CCGTGTTCTACTGCCTGAGTGTAT	GGTTCATATTACCCATGAGGCCTGGA	123	113
**ESX1L**	AGAAACTTACCGTGACCTCGCTGA	GACCCTTCCGTGCCAACGTTGTTT	112	75
**O60393**	ATCCTGACAGTGATAAACGCCGAG	CACTCCACCCTGCCACCAGTGA	102	113
**PITX3**	CCCAGAGGACGGTTCGCTGAAA	CTCTGGAAGGTCGCCTCTAGCTC	93	263
**Q9HB31**	TGGAGAGGGCGTTTGCAGCATGG	TTGGCCTCAGGAAGGCAAGTGA	85	75
**PITX1**	AGACGACCCAGCCAAGAAGAAGAA	AGCTCTTGCAACTGCTGGCTTGTGAA	72	188
**PROP1**	AGTCAGCCTTTGGGAGGAACCAGTA	CCAGTGTCCCGGGCAAGACTCTCT	67	113
**HOP**	TCCTGGAGTACAACTTCAACAAGGTCG	CGATGAGGCACAGCGTGGTGGAAT	63	225
**14**				
**DMBX1**	TTATGGTTCCCAGCACCGCAAACA	CCTCAGGCAGGTTGGTGCACAT	149	60
**RAX**	CACCGGCGAAGCGAAACTGTCAGA	GCTGTACACGTCCGGGTAGTGGGA	136	113
**OTX2**	CGTTCACTCGGGCGCAGCTAGA	CCTCGACTCGGGCAAGTTGATTT	113	113
**OTX1**	CACCACCTTCACGCGTTCACAG	GTTGATCTTGAGCGCCACCTCCT	103	68
**NP_116142**	AAGAAGAAGCACCGGAGGAACC	ACACATCCGGGTAGTGAGAGGC	94	188
**PRRX2**	CACGTTCAACAGCAGCCAACTG	CAAGCTCCTCGCGCACAAAGGC	86	113
**PRRX1**	AGCAGCGAAGGAATAGGACAACCT	GGATAGTGTGTCCGCTCAAAGACA	79	75
**PHOX2B**	CAAACTCTTCACGGACCACGGC	ACTGGTGAAAGTGGTGCGGATG	73	75
**PHOX2A**	TTCTTCCCAGAGCCATCCGGCCT	CTGGTGAACGTGGTGCGGATGC	68	94
**CRX**	CACTGTTTGCCAAGACCCAGTACC	TTGATCTTCAGAGCCACCTCCTCA	64	75
**15**				
**NKX3-1**	CTCACGTCCTTCCTCATCCAGGACA	CCGGTGCTCAGCTGGTCGTTCT	161	703
**BAPX1**	TCAGCGAGGAGAACGAGAGCAG	TTCCTCCTCTAGGTCTTTGGAAGCG	134	469
**NKX2-3**	ACTTCCACTCTGCGCCCTGCAT	ATAGGACAATTTCTCGCCCTCGTC	101	56
**HMX2**	TTCGACCTGAAGCGCTACCTGA	TGGAACCAGATCTTAACCTGCGTC	92	703
**NKX2-6**	AGTACCTGAGAATGGACGCAGAGC	AACCATCCAGCTTTCTGTCACCG	84	75
**NKX2-8**	CTGGTTCCAGAATCATCGCTACAAGC	ATGCTGCCAGGTCAGGCGACTC	77	469
**NKX2-5**	TTCACCGGCCAAGTGTGCGTCT	TCGCTGTAGGCACGTGGATAGAAGGC	66	117
**NKX2-2**	TTCTACGACAGCAGCGACAACCCGTA	TACTGAAGGCCCTCGGTGCTGG	62	117
**16**				
**LHX2**	CGGGACTTGGTTTATCACCTCAACTG	CACGTCGGCATGGTTGAAGTGTG	165	56
**LHX3**	CCCAGGACTTCGTGTACCACCT	CTCGCTGCTTGGCGGTTTCGTAGT	138	281
**LHX5**	TCATCCTGTACGGACCGCAGTTT	GTTCTGCTCCTCGTTCTCGTTGTT	126	141
**LHX1**	CCCAGTTTGTCTCCGGATTCCCAA	CCAGGTTCTGGTCGTCATTCTCGTT	115	94
**LHX9**	AAGACCAAGCGCATGCGAACCTCTTT	AAGCTGCTTGAGGTCCTTGGCAT	105	47
**LHX6**	GTTATGCAGGCGCAGTTCGCGCAG	CTGGATGACTCTCCGGCTGAGG	96	469
**GSCL**	GCCACCGCACCATCTTCAGCGA	TCGCGCGTACTCACGTCAGGATA	88	469
**GSC**	CACCAGATGCTGCCCTACATGAAC	ACAGTGCAGCTGGTTGAGAAGC	75	234

**17**					
	**TGIF2**	AACTGGTTCATCAATGCCCGGCGGCG	CTGGCCTGAGTGAAGCGGCAT	216	214
	**PBX3**	AATTGGTTTGGCAACAAACGAATCAG	GGAATTTGGTGTGGTGGGCGAA	159	161
	**C10orf48**	AATTGGTTTGCTAATGCAAGACGTCG	GAGTCATCACTGCTTACGCTAAGCCGT	137	107
	**PBX1**	AACTGGTTTGGAAATAAGCGAATCCG	GCTTCCATGGGCTGACACATTGG	120	161
	**PBX2**	AACTGGTTTGGCAACAAGAGGATTCG	CCTGGGTGACTGACACGGCG	103	107
	**PBX4**	AACTGGTTTGGCAACAAAAGAATCCG	TCCACAGCCGTTTTACCCGTGTAAAT	92	107
	**TGIF2LX/Y**	AACTGGTTTATCAATGCTCGCAGACG	TGGCCAATGATGGGGTCGTTTC	77	107
**18**					
	**EVX2**	GGATCAAGTGCGGCGCTACCGT	GGTGGTTTCGGGCAGGTTGAGT	139	107
	**IPF1**	TTCACCACCACCTCCCGGCTCA	GACTTCATCCATGGGAAAGGCAGC	127	429
	**EVX1**	CTTCACCCGAGAGCAGATTGCG	TGATGGTGGTTTCCGGCAGGTTTA	116	107
	**GSH2**	ACCCAATGGCAAGAGGATGAGGA	TTCAATCCTCCGGAGTCGAGACA	106	321
	**VENTX2**	TAAGGAGCCAAATACCTTGCGGG	GTACTGGTGGTGCTGGAAGACG	97	107
	**MEOX2**	GGACAGCATTTACCAAAGAGCAAATCAG	TATCTCGTATCGCCTCAGTCTGGT	89	107
	**MEOX1**	TGGAGGCAGAGTTTGCCCATCATA	CGCTCAGAGAGGTCCAGGTTTAC	82	107
**19**					
	**IRX1**	ACCTGGTTCGCCAACGCGCGCCGGCG	TCGCCATCGTGCTCGTCGATCT	191	125
	**IRX3**	ACCTGGTTCGCCAACGCGCGCCGGCG	CCTCCAGCTCTAGCTCGCGTTTG	163	250
	**IRX6**	ACCTGGTTCGCCAACGCACGCCGGCG	TTCTTTGGTGTCAGCAGTTAGGCAGC	139	20
	**IRX5**	ACCTGGTTCGCCAACGCGCGCCGGCG	GGGCTCGTCCTCGTCGTTCTTC	129	250
	**IRX2**	ACCTGGTTCGCCAACGCGCGCCGGCG	CGTCCTTGCTTCTGGTAGCGTCG	118	625
	**IRX4**	ACCTGGTTCGCCAACGCGCGCCGGCG	TAGGGCCGCTTCTCGTCTGCG	92	125
**20**					
	**POU4F2**	GTCTGGTTCTGCAACCAGAGGCAGAA	GCTCAGTTGTTCGCTCGTTCGC	219	250
	**POU4F1**	GTGTGGTTTTGCAACCAGAGACAGAA	CCCCAAATGCAGGCAGGATAACG	179	250
	**POU1F1**	GTTTGGTTTTGCAACCGGAGGCAGAG	TGAGGAAGAGAAAGGAATGAAACGGGAGAA	155	100
	**POU4F3**	GTCTGGTTCTGCAACCAGAGACAGAA	GATAACCATCCCCCGAACCCG	133	250
	**POU3F2**	GTTTGGTTTTGTAACAGGAGACAGAA	GAGTGTCCCTACTCCCCCCGTACA	100	250
	**POU6F2**	GTTTGGTTCTGCAATAAGAGGCAAGC	CTCCAAAGGGACTGCCGTGGC	87	125
**21**					
	**CDX1**	GGCAAAGGAGCGCAAAGTGAACAA	GCAGAAACTCCTCTTTCACAGGCA	176	68
	**BARHL2**	TTGCTGGCCGAGGCAGGGAACTA	AGGAGTCCGGTACATGCTGCTG	147	61
	**BARX2**	GAAGCTGAAGAGAAGATGAACAGCCAGG	TCTGGTGGCTCTGCCATCTCTAA	134	136
	**HLX1**	AGCGTTCTCTGCACCAAACAACAG	GCTGCTGAAGCTGAAACTATTGCC	122	102
	**HHEX**	AGGTGCTTCTTTGGATAGCTCTCAATGTTC	TTATCGCCCTCAATGTCCACTTCC	111	102
	**NANOG**	GACACTGGCTGAATCCTTCCTCT	TGGGCATCATGGAAACCAGAACAC	92	68
	**CDX4**	TTTCAGAATCGCAGAGCCAAGGAGAG	ACTTTGCACCGAGCCTCCACTATT	84	136
	**TCF2**	AGTATTCCCACACCTCCCGGTTT	CATGTTGGTGAGTGTACTGATGCTGC	77	102
	**TCF1**	TACCAGAGCTCAGACTCCAGCAAT	AAGGTCTCGATGACGCTGTGGTT	71	68
	**BARX1**	TCGGAGCCGCACTGTGTTCACC	TTCTGCTTCTCGAAGCGTTTCTCC	66	205
	**CDX2**	ATATCGAGTGGTGTACACGGACCA	GACTGTAGTGAAACTCCTTCTCCAGC	62	153
**22**					
	**SIX5**	CTCCATCCTGGTGAACGGGAGCTT	AGAGAGGTCTTGGTCTCGCTGG	195	68
	**SIX4**	ACTGGTCAAGACCTATTGTCAGTCCC	GTTAGCTACCGATTGCAGAACCAAACG	150	61
	**SIX6**	CTCCAGCAGCAGGTCCTGTCAC	ATGGAGATGGCTGAAGTGGCCG	137	55
	**SIX2**	GAGAACAACGAGAACTCCAATTCTAACAGC	GGGCTGGATGATGAGTGGTCTG	125	85
	**SIX1**	TCCTCCTCCAACAAGCAGAACCAA	AAGGACCGAGTTCTGGTCTGGACTTT	114	102
	**PKNOX2**	ATGCTTGATGCCAGCAACCCAGAT	GCGATGGAGTTGGGCCAGAATCTTT	95	68
	**MEIS2**	CACCCTGGAATGACTATGTCAGCA	ATGAATGTCCATAACCTGTCCGCC	87	136
	**PROX1**	GCATTACCTCTCAGCTCATCAAGTGG	TTGACGTGCGTACTTCTCCATCTG	80	136
	**MEIS1**	GCCACCGGTATATTAGCTGTTTGAAAGGG	TGATCCTCCTTCTCTATCGTCTATCACC	74	170
	**PKNOX1**	ACGTGGACAGCCTTCAGTCTCTGT	CTGCCATCATGACCTGCTGCAC	69	68
	**MEIS3**	CTCTCCCAGTTCTGGTGGAGAAGAT	CCCTCTTCTTGTTTCGCCGTCGCT	65	102
**23**					
	**CDX2**	ATCTGGTTTCAGAACCGCAGAGCAAA	TGCAGGGAAGACACCGGACTCA	173	83
	**TCF1**	AACTGGTTTGCCAACCGGCGCAAAGA	CCTTACTGGGGGAGAGGGCAGGT	154	83
	**HLX1**	GTGTGGTTCCAGAACCGGCGGATGAA	GGCTGGGGCTCCTCTCGTCCTGCTCG	142	50
	**CDX1**	ATCTGGTTCCAAAACCGGCGGGCAAA	GGCCCCCCAGGGATGGCCCG	130	83
	**CDX4**	ATCTGGTTTCAGAATCGCAGAGCCAA	CTGATGGAGTCAGAGTCACTTTGCACCGAGCCTCC	107	83
	**HHEX**	AGGTGAGATTCTCCAACGACCAGA	ATCTTGGCCAGACGCTTCCTCT	97	250
	**TCF2**	AACTGGTTTGCAAACCGCAGGAAGGA	CAGGCTGTGAGTCTGGTTGGAGCTATAG	87	67
	**NANOG**	ACCTGGTTCCAGAACCAGAGAATGAA	TGCGTCACACCATTGCTATTCTTC	80	125
	**HHEX**	TGTGATCAGAGGCAAGATTTGCCCAGTGAA	GGGGAGGGCGAACATTGAGAGCTA	77	208
**24**					

	**IRX3**	AGGGAAACGCTTATGGGAGCGA	GCGCCGTCTAAGTTCTCCAAATCG	181	250
	**IRX1**	GCTCGCAGTATGAACTGAAGGACA	CTTGAGCGTGCTGGTGCTCTCG	152	250
	**IRX5**	ACACGAACTATGGCTCCTTCGGA	TTCGGGTCTTTAGCCAAAGCGT	139	417
	**TGIF2LX/Y**	ACAGGCAGAGTTCTTGCCTTACCA	CTTCTTCTGAAGGGTAGGCCTTAAACCG	127	188
	**TGIF2**	AGCAGGACAAAGAGGACTTCAGCA	AGTGTGCAGTAATGGGAGTGATGG	116	104
	**C10orf48**	CAAGAGCAGCTTGTTGAACCGTTACC	AGATCCCGAGTGGTTTCTTTGCCT	106	83
	**IRX6**	CGAATCTACCCAACGCTCTGTCTCA	ACTGCCCAGCAGTCGACTATCGTA	97	208
	**IRX4**	GCTGCAGGAGCACCGCAAGAAC	AGACCTGTGTGAGGGTCATCTTGGT	89	83
	**TGIF**	TTCCCTTGGACCTTTCTTCATCCG	AGAATCTGCACAGACTCCTTGGGT	82	125
**25**					
	**CHX10**	GTCTGGTTCCAGAACCGTCGAGCCAA	TGGCTGACTTGAGGATGGACTCG	142	188
	**ALX4**	GTCTGGTTCCAGAACCGAAGGGCCAA	TCATATGCAGTGGAGAAGTGGGTTCG	92	125
	**ALX3**	GTCTGGTTCCAGAACCGCAGAGCCAA	CAGCCGTGAAGGGGTTCCGCCCCTC	85	100
	**VSX1**	GTCTGGTTTCAAAACCGCAGGGCCAA	CGTACTCGGCCATCACGCTGCT	79	188
	**CART1**	GTTTGGTTTCAAAATCGAAGGGCCAA	GCTTTTCGCTTGTTGTATTTGGCCATAAC	72	125
	**HESX1**	ATTTGGTTTCAAAATCGGCGTGCAAA	CGCCATTAGAAACTGTGATTCTCTATGGG	66	188
**26**					
	**POU6F1**	GTCTGGTTCTGCAATCGGCGCCAGAC	GATGCACATGCACACGGTGGA	215	300
	**POU3F4**	GTCTGGTTCTGTAATCGAAGACAAAA	TGGCAAGATGTGTCTGTTTTCACGG	107	600
	**POU2F2**	GTCTGGTTCTGCAACCGGCGCCAGAA	CTGTAGCTGGCCGGCTTCCCT	95	120
	**POU2F1**	GTTTGGTTCTGTAACCGCCGCCAGAA	GCTTTAATAGGTGAGCTGCTGGTCCCAC	83	150
	**POU2F3**	GTCTGGTTCTGCAACCGACGCCAAAA	TGTAGACAGGTGGTTTGATGGGTGTG	79	150
**27**					
	**EN1**	ACCAAATACCCGGAGCACGGCAA	GGACGATCCGAATAACGTGTGCAGTA	137	125
	**NP_689781**	ACCTGGGTCTAGGCTTTGCTCA	ATGCAGGAGTGTCTGCTTCTTCC	125	83
	**EMX2**	AGTCGCTGGTGGCCAAGGACAG	AGCCGTTGAGGAACGGATTTATGG	114	417
	**NP_940881**	TTGCCAGACACCCAGTTATTCCCT	CCTTCTTGGTACTGAGAGGTCATGGT	104	104
	**VAX2**	GAAATTGTCCTGCCTAAGGGCCTG	AACTCCATCTCCAGGCGGTACA	95	167
	**VAX1**	GCTCTATCGGCTGGAGATGGAGTT	GAGAGGTTAAGCTGCCGGGCGA	87	333
	**EMX1**	AGCGCGCCTTCGAGAAGAACCACTA	CGTCTCGGAGAGGCTGAGACTG	80	104
	**NKX6-2**	AACCTTCGAGCAGACCAAGTACC	TCTCGGTCATGCCCAGCGAGTA	74	625
	**NKX6-1**	GCCCTGGAGAAGACTTTCGAACAA	CGAATAGGCCAAACGAGCCCTCT	69	167
